# MTH1 as a Chemotherapeutic Target: The Elephant in the Room

**DOI:** 10.3390/cancers9050047

**Published:** 2017-05-08

**Authors:** Govindi J. Samaranayake, Mai Huynh, Priyamvada Rai

**Affiliations:** 1 Department of Medicine/Division of Hematology and Oncology, University of Miami Miller School of Medicine, Miami, FL 33136, USA; gjs26@med.miami.edu (G.J.S.); c.huynh@miami.edu (M.H.); 2 Sheila and David Fuente Graduate Program in Cancer Biology, University of Miami, Miami, FL 33136, USA; 3 College of Arts and Sciences, University of Miami, Coral Gables, FL 33146, USA; 4 Sylvester Comprehensive Cancer Center, Miami, FL 33136, USA

**Keywords:** MTH1, oxidative stress, cancer, therapeutic target, nucleotide pool, p53, RAS oncogene, MTH1 inhibitors

## Abstract

Many tumors sustain elevated levels of reactive oxygen species (ROS), which drive oncogenic signaling. However, ROS can also trigger anti-tumor responses, such as cell death or senescence, through induction of oxidative stress and concomitant DNA damage. To circumvent the adverse consequences of elevated ROS levels, many tumors develop adaptive responses, such as enhanced redox-protective or oxidatively-generated damage repair pathways. Targeting these enhanced oxidative stress-protective mechanisms is likely to be both therapeutically effective and highly specific to cancer, as normal cells are less reliant on such mechanisms. In this review, we discuss one such stress-protective protein human MutT Homolog1 (MTH1), an enzyme that eliminates 8-oxo-7,8-dihydro-2’-deoxyguanosine triphosphate (8-oxodGTP) through its pyrophosphatase activity, and is found to be elevated in many cancers. Our studies, and subsequently those of others, identified MTH1 inhibition as an effective tumor-suppressive strategy. However, recent studies with the first wave of MTH1 inhibitors have produced conflicting results regarding their cytotoxicity in cancer cells and have led to questions regarding the validity of MTH1 as a chemotherapeutic target. To address the proverbial "elephant in the room" as to whether MTH1 is a bona fide chemotherapeutic target, we provide an overview of MTH1 function in the context of tumor biology, summarize the current literature on MTH1 inhibitors, and discuss the molecular contexts likely required for its efficacy as a therapeutic target.

## 1. Introduction

Human MutT Homolog 1 (MTH1 a.k.a Nudix hydrolase 1, NUDT1) is an 18 kD Nudix pyrophosphatase that hydrolyzes the oxidized purine deoxyribonucleotides, 8-oxo-dGTP, as well as the less frequently-occurring 2-OH-dATP and 8-oxo-dATP, and their ribonucleotide analogs into their monophosphate equivalents [1,2,3,4]. These enzymatic byproducts are then targeted for degradation to prevent their re-use by deoxyribonucleotide salvage pathways [3]. This function is essential for preventing incorporation of such oxidized precursors into genomic DNA [5], as many repair or translesion DNA polymerases exhibit poor discrimination between undamaged and oxidatively-damaged dGTP [6,7] and replicative polymerases show comparable rates of dCTP insertion opposite 8-oxodG vs. dG [8]. As genomic 8-oxoguanine (8-oxoGua) repair entails production of DNA backbone nicks, frequent initiation of this base excision repair (BER) process via the 8-oxoGua-excising enzymes such as 8-oxoGua glycosylase 1 (OGG1) can potentially produce genomic instability [9,10,11]. MTH1 also hydrolyzes oxidized purine ribonucleotides, although ribonucleotide reductase (RNR) is unable to utilize 8-oxo-GTP as a substrate and, hence, cannot be used as a substrate for 8-oxodGTP formation [12]. However, the importance of MTH1 function in RNA quality control may lie in alternate nucleotide salvage and anabolic pathways that enable incorporation of 8-oxoGua in RNA synthesis [13]. Although MTH1 has been reported to hydrolyze 2-OH-dATP more efficiently than 8-oxo-dGTP [14], the high intracellular abundance of 8-oxo-dGTP [5] makes the functional 8-oxo-dGTPase activity of MTH1 more physiologically relevant to biological systems. Despite this critical role in cell function, the *MTH1* germline knockout mouse is developmentally normal and displays no pathological phenotypes, barring low incidences of spontaneous late age-onset lung, liver, and stomach tumors [15]. Treating the *MTH1*-null mouse, the *MTH1*-null/*OGG1*-null mouse, or mouse embryonic fibroblasts (MEFs) derived from these animals with oxidant-generating chemicals such as the mitochondrial poison 3-nitropropionic acid or hydrogen peroxide has been reported to increase mitochondrial DNA oxidation and induce neurotoxicity or general cytotoxicity [16,17,18]. Thus, the functional requirement for MTH1 emerges when cells sustain elevated ROS levels, presumably through ensuing increased production of oxidized substrates in the nucleotide pool. In the absence of such oxidative stress, under normal physiologic conditions, MTH1 loss does not produce deleterious effects, suggesting either a low level of oxidatively-generated damage in the nucleotide pool or efficient removal of 8-oxoGua from genomic DNA through repair pathways, or both. 

## 2. A Functional Role for MTH1 in Tumor Biology

Unlike normal cells, tumors produce high levels of oxidants due to chronically-hyperactivated mitogenic and pro-survival signaling, as well as metabolic alterations [19]. MTH1 protein expression is found to be higher in tumor tissue relative to matched normal tissue [20,21,22] suggesting that the tumors have a greater reliance on MTH1 function, presumably due to their higher levels of oxidative stress. In non-small cell lung carcinoma (NSCLC), a tumor type characterized by dysfunctional BER and low levels of OGG1 [23,24], MTH1 has been reported as the major determinant of genomic 8-oxoGua levels and is associated with low levels of G -> T transversions [25], a signature 8-oxoGua mutation due to its enhanced ability to pair with adenine. Our studies, along with others, have shown MTH1 protein levels are elevated in NSCLC patient-derived tumors, when compared to adjacent normal lung tissue [26,27].

Due to its canonical anti-mutator function, the prevailing notion was that MTH1 plays a tumor-suppressive role in cells, and that the elevated late-onset spontaneous tumor formation observed in *MTH1*-null animals is a result of increased mutagenic burden in the *MTH1*-null mouse [5,15]. However, it has been subsequently shown that *MTH1*-null animals do not possess elevated levels of the hallmark G -> T tranversions expected due to increased genomic 8-oxoGua incorporation [28] and, in fact, their tumor incidence is abolished in the *OGG1*-null background [29]. Thus, the tumors in *MTH1*-null mice likely arise through alternate mechanisms, potentially through MTH1 inhibition-induced genomic instability in the setting of aging-associated oxidative stress. Consistent with this idea, we were the first to show the acute effects of MTH1 inhibition in human cells are irreparable genomic strand breaks that are capable of inducing p53-dependent cell senescence under high oxidant, but not low oxidant, environments [30]. Research from our laboratory in the last few years has further shown MTH1 is a critical player in oncogenic RAS-driven tumorigenesis and that its elevated expression facilitates the spectrum of oncogenic RAS-driven transformation, as follows. We reported MTH1 overexpression prevents accumulation of oncogenic RAS-induced oxidatively-generated DNA damage, persistent double strand breaks (DSBs), and ensuing oncogene-induced senescence (OIS), which is the first barrier to transformation in normal cells [31]. We then went on to demonstrate that elevated MTH1 levels facilitate transformation of immortalized cells via oncogenic RAS and enable maintenance of multiple tumor-promoting phenotypes, such as anoikis resistance and epithelial-mesenchymal transition (EMT), that rely on ROS-producing pathways, e.g., Akt or Rac1 signaling [26,32]. Conversely, we showed knocking down MTH1 in NSCLC cells with activating KRAS mutations reduces their tumor formation ability and eliminates the highest RAS oncoprotein-expressing subpopulations in the tumor, leading to an overall reduction in ROS levels, as well as Akt signaling [27]. Importantly, NSCLC cells that possessed wild-type KRAS were not susceptible to MTH1 inhibition-induced proliferative defects or strand breaks unless the constitutively-active oncogenic variant KRASV12 was introduced into these cells [27]. Based on our collective body of work we, therefore, concluded MTH1 functions as a non-oncogene addiction [33], an inherently non-tumorigenic pathway that promotes malignancy, without which oncogenic RAS-harboring cells lose their pro-tumorigenic abilities [34]. Congruent with this idea, we showed *MTH1* mRNA levels positively correlate with *KRAS* levels, even in early-stage human NSCLC tumors, and that introduction of oncogenic RAS is sufficient to upregulate MTH1 mRNA and protein expression [27].

**Figure 1:**
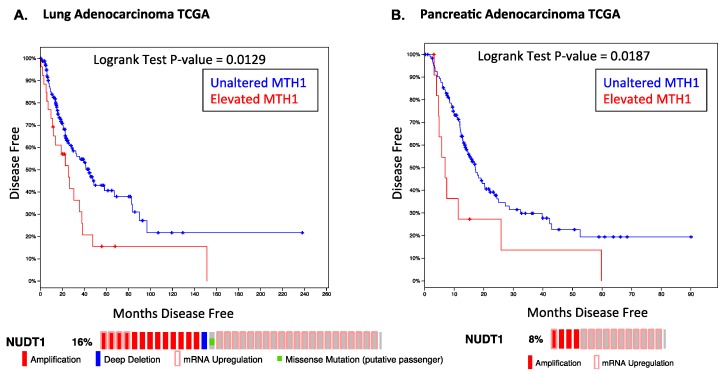
Effect of elevated *MTH1* mRNA levels on disease-free survival in RAS-driven cancers. Data and statistics were obtained from the cBioportal website for the indicated TCGA provisional datasets. The percentage of the total samples in which *MTH1* (*NUDT1*) gene is amplified or mRNA is upregulated is shown below the survival curves. (**A**) Lung adenocarcinoma dataset. Kaplan-Meier curve data represent 221 cases. Median months disease-free (*NUDT1* unaltered) = 44.02, median months disease-free (*NUDT1* elevated) = 25.33; (**B**) Pancreatic adenocarcinoma dataset. Kaplan-Meier curve data represent 141 cases. Median months disease-free (*NUDT1* unaltered) = 17.12, median months disease-free (*NUDT1* elevated) = 6.93.

Consistent with our results, meta-analyses of public datasets indicate that RAS-driven tumors, such as pancreatic cancer or lung adenocarcinoma, show poor disease prognosis and higher relapse rates in tumors where MTH1 is elevated (Figure 1). Notably, MTH1 is largely either amplified or overexpressed in these tumors, rather than being mutated or deleted, indicating it would be an actionable target. As targeted inhibition of oncogenic RAS has been unsuccessful thus far in the clinic, our work indicates that targeting MTH1 in lieu of RAS could present an alternative approach to eliminating oncogenic RAS-harboring tumor cells or other oncogene-driven tumors that possess elevated ROS levels [34].

## 3. Outcomes of MTH1 Inhibitors in Different Cancer Models

Building on our observations regarding the role of MTH1 in preventing genomic DNA breaks and OIS [30,31], the first-in-class inhibitors against MTH1 were recently reported [35,36] and recapitulated our initial findings that MTH1 inhibition was sufficient to produce DNA breaks and induce tumor suppressor responses. These reports were followed by additional studies describing the effects of additional small-molecule MTH1 inhibitors or the targeted inhibition of MTH1 via shRNA, siRNA, or CRISPR in a wide variety of cancer cell lines [37,38,39], ostensibly producing variable and inconsistent outcomes with regards to cell viability, often in the same cell lines (models, methodology and outcomes are summarized in Table 1).

The first-in-class MTH1 inhibitors, TH287 and TH588, reported in 2014 [35], consist of an aminopyrimidine moiety that binds the MTH1 active site at its Asn33, Asp119, and Asp120 residues, thus abrogating its 8-oxo-dGTPase and 2-OH-dATPase activity. These inhibitors were tested in a panel of well-known cancer cell lines, via clonogenic survival assays and subcutaneous xenograft tumor models in immunocompromised mice (see Table 1), and were found to reduce viability and tumor volumes to various degrees in all the lines tested. These studies noted TH287 was a more potent MTH1 inhibitor than TH588. Molecular characterizations of inhibitor treatment outcomes were carried out in a more limited panel of cell lines, mainly in U2OS cells, with additional proof-of-concept testing with an MTH1 siRNA in HeLa cells. These studies showed the inhibitors activated Rad51 and DNA-PKcs-mediated DNA repair pathways, and (similar to our earlier study with MTH1 shRNA knockdown) [30] produced irreparable genomic DNA double-strand breaks (DSBs) marked by 53BP1 foci and upregulated p53 levels, leading to cytotoxicity. This study also showed that siRNA-mediated depletion of MTH1 increased total cellular 8-oxoGua (measured via fluorescently tagged-Avidin), increased genomic 8-oxo-dG incorporation (as measured by a modified OGG1/MUTYH comet assay), increased 53BP1 foci, and reduced survival of U2OS cells. Expression of the RNAi-resistant wild-type MTH1, but not the catalytically dead MTH1 protein (E56A), decreased 8-oxo-dG levels and 53BP1 foci, and led to reduced cytotoxicity under siMTH1. While this study reported mainly cell death markers with MTH1 inhibitors and senescence only with siRNA (using H3K9Me3, but not more usual senescence markers [40]), our study [27] and another study in which MTH1 was downregulated through miR-145 depletion [41], did not observe MTH1 inhibition-induced cell death, but rather only anti-proliferative effects. This discrepancy may reflect the effects of genetically-depleted MTH1 versus pharmacologic MTH1 inhibition. 

The (S)-enantiomer of crizotinib, a well-known Met/ALK inhibitor, was also reported in 2014 as a nanomolar suppressor of MTH1 activity [36]. By contrast, the (R)-enantiomer only inhibited MTH1 catalytic activity in the micromolar range. Similar to the TH inhibitors [35] and our prior studies with MTH1 knockdown [30], MTH1 inhibition via (S)-crizotinib led to an increase in immunofluorescent staining for DSB-specific markers, such as 53BP1 and autophosphorylated ATM, in human colon carcinoma cells. Treatment with (S)-crizotinib also led to increased DNA tail moments in the alkaline comet assay that were further enhanced upon treatment with OGG1 and MUTYH, thus supporting the idea that (S)-crizotinib led to elevated genomic 8-oxodG. Treatment with (S)-crizotinib, but not (R)-crizotinib, also reduced xenograft tumor formation by the SW480 colorectal cancer cell line.

Following these first-in-class inhibitors, several other groups published studies describing their own MTH1 inhibitors. Kettle et al. [38] at Astra Zeneca tested TH588 (but not the more potent MTH1 inhibitor, TH287) and (S)-crizotinib against their own MTH1 inhibitors in a comparative study against three factors: ability to inhibit MTH1 enzymatic activity (using release of inorganic pyrophosphate, iPP), binding affinity of the inhibitors to the MTH1 protein and half-life for dissociation (via surface plasmon resonance to obtain Kd values). They also carried out a target engagement assay, to assess off-target effects of their inhibitors (using a whole cell thermal stabilization assay (CETSA) to generate EC50 values). They established MTH1 IC50 vs. CETSA EC50 values to correlate inhibitor potency against target engagement for each inhibitor being evaluated. Two of their small molecule inhibitors (compounds **15** and **19**) showed better potency and engagement vis-à-vis TH588. (S)-crizotinib, by contrast, showed much weaker target engagement in their comparison. This study also evaluated the effects of MTH1 depletion via siRNA and CRISPR knockout on U2OS and SW480 cells, respectively. Unlike the studies of Gad et al. and Huber et al. [35,36], they found that SW480 cells, a double-mutant p53 line with mutated KRAS, did not show a growth defect in clones lacking MTH1 via CRISPR and concluded that MTH1 is not required for viability in this cell line. It should be pointed out that Huber et al. and Gad et al. used shRNA and siRNA to deplete MTH1 in these cells [35,36]. Kettle et al. [38] also treated U2OS cells with TH588 or their compound **19** and assessed a panel of DNA damage markers via Western blotting (see Table 1). They found that whereas TH588 (denoted as compound **1** in their study) induced the elevation of pSer15-p53, total p53, and cleaved PARP1, re-capitulating the original results with this compound [35], their own compound produced minimal changes in these markers. These molecular changes were not assessed under shRNA- or CRISPR-mediated MTH1 depletion in their study.

While these results [38] may seem to contradict a role for MTH1 in cancer cell viability, several factors need to be taken into consideration. In particular, it should be noted that one irreparable DNA DSB in a cell is sufficient to produce tumor-suppressive outcomes [43] and that MTH1 inhibition (unlike radiation or other extrinsic stresses) produces a small number of such DSBs in a cell [27,30]. Thus, immunoblotting for protein levels may not be sensitive enough to robustly detect changes in DNA damage markers, such as gammaH2AX (γH2AX) or RPA levels, stemming from such small numbers of DSBs. Detecting MTH1 inhibition-induced DSBs typically require single cell-based assays, such as the alkaline single cell gel electrophoresis (“comet”) assay or staining for DSB foci [27,30,35]. Consistent with this notion, this study [38] does not find changes in γH2AX levels by immunoblotting, even with TH588 (compound **1**) treatment, which generated γH2AX-containing DSB foci in the original study [35]. Interestingly, both TH588 and their compound **19** elevated total p53 levels (indicating induction of a genotoxic response) and, although cleaved PARP levels were observed only for TH588 (compound **1**), they did not assay for proliferation defect markers such as p21^cip1^ or p27^kip1^, which can be induced downstream of p53 and may have occurred with their compound **19**. The use of population-based, rather than single cell, assays renders their results inconclusive rather than negative. They also observed no effects on viability with a commercial siRNA against MTH1, but were able to do so with the siRNA sequence used by Gad et al. [35]. In and of itself, this phenomenon is not surprising as no siRNAs against the same target are equally effective [44]. Thus, the lack of consistent effects between their siRNA target sequence and the one used by Gad et al. [35] is not conclusive evidence for the Gad et al. siRNA producing off-target cytotoxicity. Our own studies [26,27,30,31] have used completely distinct target shMTH1 sequences from the one described in Gad et al. [35] and found them to produce consistently negative effects on DSBs and cell proliferation. 

Kettle et al. [38] treated MTH1 siRNA-transfected U2OS cells with TH588, (S)-crizotinib, and their compound **19**, and found all three compounds killed cells transfected with the siMTH1 sequence used by Gad et al. [35]. They concluded this observation supported the non-MTH1 suppression-associated toxicity of this siRNA sequence. While this is certainly one possibility, key experimental details about the conditions under which these results were obtained are lacking. For instance, both SW480 and U2OS are extremely fast-growing cell lines and we have repeatedly found there is a strong selective pressure on the initially small population of cells that escape MTH1 inhibition (through suboptimal genetic suppression or poor drug response/uptake) in such rapidly proliferating cell lines (e.g., H460 [27]). Thus, the non-MTH1 inhibited population can overtake cultures within a few days [27]. Without a clear assessment of MTH1 status in the cultures at the end, as well as the beginning, of their proliferative or colony-forming experiments, these experiments may well represent outcomes from a selective cell population that either did not sustain MTH1 loss or has lost the suppressive element. Nevertheless, these results also raise the possibility that there are alternate mechanisms able to compensate for the loss of MTH1 activity, or that the TH588 and (S)-crizotinib inhibitors, like many drugs, may produce off-target effects along with on-target cytotoxicity. 

According to proteomics profiling carried out by Kawamura et al. [37], the TH588 and TH287 inhibitors appear to have a profile similar to microtubule inhibitors, such as vinblastine, suggesting off-target cytoskeletal degradation stress-associated cytotoxicity. However the concentrations of inhibitors used in these studies are extremely high (30 µM) compared to the effective range (10 µM or less) established by the originating studies [35] in their culture models. The Kawamura et al. study also described their in-house MTH1-specific inhibitors (NPD7155 and NPD9948), and reported minimal effects on viability and DNA damage markers in several pancreatic cancer cell lines (the p53- and KRAS-mutant MiaPaCa-2 and PANC-1 lines), as well as HeLa and Jurkat cells, which have a tendency to accumulate p53 mutations or deletions in culture. We have comprehensively shown that p53-nonfunctional cells exhibit much smaller effects of MTH1 inhibition in vitro than in vivo, and also do not undergo DNA breaks [27]. Kawamura et al. also reported that murine NIH 3T3 cells transformed with KRASV12 did not show selective sensitization to their MTH1 inhibitors. This result needs to be interpreted with caution given that both antioxidant mechanisms [45] and transformation mechanisms [46] are very different in murine vs. human cells. Additionally, an MTH1 inhibitor targeting the human protein is not expected to show the same degree of efficacy against the murine homolog [35]. As proof-of-principle, Kawamura et al. also depleted MTH1 via siRNA in a single fast-dividing cell line (HeLa) and assessed the effects on cell viability. The same caveats described above for the Kettle et al. [38] study, utilizing genetic MTH1 depletion in rapidly growing cells, holds true here as well. Thus, their claims regarding the dispensability of MTH1 function in cancer following the assessment of MTH1 inhibition in such a limited panel of cell lines (mostly lacking functional p53), and without any in vivo characterizations of MTH1 inhibition, are likely premature. 

Petrocchi et al. at MD Anderson also reported their in-house MTH1 inhibitors, IACS-4619 and IACS-4759 [39]. Both of these compounds apparently inhibited MTH1 activity more effectively than TH588 and TH287 (with IC50 values 2.5–8 times lower than TH287). The IACS compounds also exhibited good cell permeability and solubility. IACS-4579 was found to be stable in rat and human plasma and liver microsomes, whereas IACS-4619 showed good permeability, but high turnover, in liver microsomes. These inhibitors were tested in vitro in a large number of human cancer and normal cell lines (U2OS, SaOS2, SW480, HCT116, MDA-MB-231, HeLa, UOK262, 293T, A549, H460, H358, WI-38, BJ, hMEC, MRC5), and apparently did not produce any cytotoxicity or anti-proliferative effects at concentrations up to 50 µM. However, neither these data, nor experimental details under which they were obtained, are explicitly included in the publication, nor are any molecular or in vivo tumorigenicity data presented in support of their conclusions. This study, however, does report a cytotoxic phenotype for TH287 and TH588 at micromolar concentrations in cell lines with high ROS levels, consistent with the original observations with these compounds [35]. 

Recently, Helleday and colleagues published their best-in-class inhibitor, TH1579, which was able to reduce viability in a number of cell lines, concomitantly elevating p53 and DNA DSB markers [42]. In this comprehensive study, Warpman et al. examined the selectivity profile of TH1579 against 87 other proteins besides MTH1, including kinases and base excision repair proteins. They also assessed mRNA (but not protein) levels of related Nudix proteins, such as OGG1, MTH2, MYH, and NUDT5, along with MTH1, and found none of these were altered upon inhibitor treatment. Thus, this inhibitor showed very high on-target specificity. This study also attempted to reconcile some of the disparate and conflicting results in the above-discussed publications. They utilized the siMTH1 sequence used by Kettle et al. [38] (denoted as AZ siMTH1) and found it, indeed, induced cytotoxicity, in contrast to the observations reported by Kettle et al. However, they tested this siRNA in NTUB1/P, rather than U2OS, cells used by Kettle et al. [38]. Thus, the same MTH1 siRNA apparently produced different effects in different cell lines, alluding to the potential of context-specific effects arising from MTH1 inhibition. 

Warpman et al. [42] also carried out proteomic profiling of their inhibitors, to compare their results against those of Kawamura et al. [37] However, they found their compounds clustered with nutrient starvation rather than microtubule inhibitors as reported by Kawamura et al. [37]. The source of this inconsistency between the two studies could be due to the different cell lines (HeLa [37] vs. HCT116 [42]), drug doses and time points (TH287 at 3 µM for 18 h [37] vs. TH588 at 5 µM for 72 h [42]) and, potentially, the actual methods used for proteomic profiling. Finally, Warpman et al. reported that, unlike TH588, several of the MTH1 inhibitors that did not induce cytotoxicity [38,39] also did not elevate 8-oxo-dGTP incorporation into genomic DNA, a critical factor in order for MTH1 inhibition to induce DNA breaks and tumor suppressor responses [30]. However, given the relative potencies of the other MTH1 inhibitors, some of which are able to inhibit MTH1 activity as well as, or better [39] than, TH588 and TH287, it is unclear why MTH1 inhibition by these compounds is unable to elevate oxidized nucleotides and their subsequent incorporation into genomic DNA. This issue (and other paradoxical and conflicting observations discussed above) can only be resolved if there is greater consistency among these studies across multiple experimental parameters, including drug concentrations, siRNA sequences, molecular profiling methodology, and common authenticated cell lines.

## 4. Molecular and Cellular Contexts Underlying the Outcomes of MTH1 Inhibition

Taken collectively, the reported discrepancies among recent studies characterizing MTH1 inhibitors in cancer models reinforce the idea (supported in part by our findings) [26,27] that MTH1 inhibition-associated outcomes rely on several key molecular factors. Foremost among these is whether there is a strong oxidant driver present, either oncogenic RAS (as we have shown) [26,27] or some other ROS-producing cancer-relevant mechanism, such as Akt hyperactivation or inflammation, leading to the chronically-elevated ROS levels required to produce sufficient levels of MTH1 substrates in the nucleotide pool, to evoke an integral role for MTH1. Additional critical factors affecting MTH1 inhibition response are likely to include the efficiency of DNA maintenance and repair pathways employed by different cancer cells. Caglayan et al. recently showed that the introduction of oxidized nucleotides by DNA polymerase beta affects the critical ligation step of base excision repair, producing toxic DNA breaks, and that MTH1 inhibition enhances this effect by increasing the availability of oxidized DNA precursors [47]. Similarly, Fouquerel et al. showed that MTH1 depletion in telomerase-positive cells with shortened telomeres produces telomere dysfunction and cell death, and suggested that elevated 8-oxoGua levels can subvert normal telomerase activity in maintaining telomere length and structure by inhibiting telomere extension [48]. We have shown, in the absence of wild-type p53, that MTH1 inhibition can induce tumor suppression without producing DNA strand breaks, by promoting the loss of tumor cells with high levels of ROS-driven oncogenic signaling through an as-yet undefined mechanism [26,27]. Another critical issue which remains to be rigorously addressed in the therapeutic inhibitor studies is whether the genomic 8-oxodG levels measured by the modified comet assay indeed reflect incorporation from the oxidized nucleotide pool or are produced in situ. The latter situation could occur if the inhibitor in question raises cellular ROS as an off-target effect, leading to oxidatively-damaged genomic DNA and artifactually mimicking an expected on-target effect of MTH1 inhibition. 

Cancer cells are characterized by metabolic plasticity, which affects the cellular redox state, as well as ROS production. In this regard, it was recently reported that the loss of the Von-Hippel Lindau (VHL) factor, a tumor suppressor and E3 ubiquitin ligase that degrades the hypoxia-inducible factor HIF under normoxic conditions, sensitizes cells to MTH1 inhibition [49]. The unaltered mutagenic burden in MTH1-null mice [28] and the lack of difference in genomic 8-oxodG processing byproducts, such as 8-oxo-7,8-dihydro-2′-deoxyguanosine, in *MTH1-null* vs. *wild-type* animals [50] potentially point to redundant mechanisms that can compensate for MTH1 loss. Alternatively, enhanced antioxidant pathways, such as Nrf2 [51], could mitigate the need for MTH1 function by reducing ROS levels and, thus, oxidation in the nucleotide pool. We previously showed that MTH1 suppression via shRNA induces oncogene-induced senescence (but not cell death) in RAS-driven, p53 wild-type NSCLC cells [27]. Thus, alternate tumor suppressor pathways, such as senescence, necrosis, or autophagy, as well as the status of the molecular tumor suppressors mediating these pathways, need to be evaluated under MTH1 inhibitor treatment. The subcellular localization of MTH1 may also be critical, as it has been shown to be present in the mitochondria [5] and may protect against electron transport chain-induced ROS through preventing mitochondrial genome DNA damage. Furthermore, very little is currently known regarding post-translational modification, cell cycle-specific context, or spatiotemporal control of MTH1 function, all of which could affect the outcomes of MTH1 inhibitors and the model systems utilized. 

Finally, we note that a key difference between the studies that show a tumor-suppressive effect of MTH1 inhibition (including ours) [27,35,36,42] and those that do not [37,38,39] is that the former studies explicitly test effects of MTH1 inhibition in in vivo tumor formation models, whereas the latter solely utilize in vitro models (see Table 1). We have shown that a fairly modest in vitro proliferative defect (particularly in p53-nonfunctional cell lines, which many of the conflicting studies utilize) can manifest as a strong defect in in vivo tumor formation [27]. Thus, assessing effects of MTH1 inhibitors on in vivo tumorigenic models is likely the single most critical factor in determining their potential translational utility.

## 5. Conclusions

**Figure 2:**
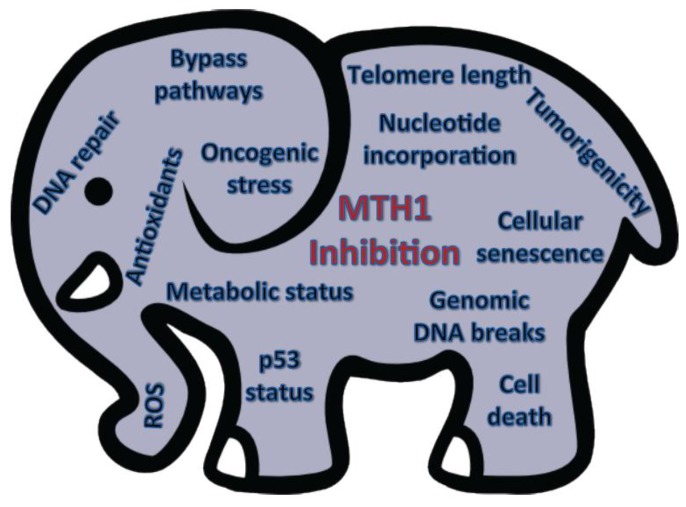
The elephant in the room. Schematic summarizing the molecular and cellular contexts that likely affect MTH1 inhibition-associated outcomes.

In conclusion, the role of MTH1 biology in tumors is still in its nascent stages and the utility of its therapeutic inhibition, the proverbial current “elephant in the room”, cannot be evaluated without a comprehensive understanding of the complex molecular factors that governs its function in tumor cells (Figure 2). While the various cancer-relevant studies on MTH1, thus far, have all provided valuable information, its importance as a therapeutic target will only become apparent when these studies are collectively placed in a context designed to provide consensus among the results acquired in different model systems. 

## Figures and Tables

**Table 1:** Summary of current MTH1 inhibitors and their outcomes.

Publication	Cell Line	Cell Type	Mode of MTH1 Inhibition	Mode of Measurement	Outcome
Gad et al., Nature, 2014 [35]	U2OS	Human osteosarcoma	siRNA, TH287, TH588	-Cell viability, clonogenic survival -Immunofluorescent staining (8-oxoGua via avidin-AlexaFluor488 staining , 53BP1, RPA, phospho-S1981 ATM, RAD51, phospho-S2056 DNA-PKcs, cleaved caspase 3, H3K9me3) -OGG1/MUTYH alkaline comet assay -Immunoblot p53 (S15), p21cip1, phospho-S1981 ATM	-siRNA against MTH1: decreased cell viability and clonogenic survival, increased total cellular 8-oxoGua, RPA, 53BP1 foci, H3K9me3, and cleaved caspase 3. DNA damage was induced as measured by RAD51, DNA-PKcs mediated DSB repair, increased ATM-dependent (S15) phosphorylation, p21cip1. Increased OGG1/MUTYH comet tail moment showing increased 8-oxo-dG incorporation into DNA -TH588 or TH287: increased total cellular 8-oxoGua, elevated cytotoxicity, DNA damage (induced 53BP1, DNA-PKcs foci, increased OGG1/MUTYH comet tail moment showing increased 8-oxo-dG incorporation into DNA)
	VH10	Primary cells, human foreskin fibroblasts	siRNA, TH287, TH588	-Cell viability, clonogenic survival -Immunofluorescent staining (8-oxoGua via avidin-AlexaFluor488 staining , 53BP1, RPA, phospho-DNA-PKcs) -Immunoblot (p53 (S15), p21cip1, phospho-S1981 ATM	-No decrease in clonogenic survival nor increase in total cellular 8-oxoGua with MTH1 siRNA -No 53BP1, RPA, DNA-PKcs foci with TH287, TH588 -No change in ATMpS1981, p53 (S15), or p21cip1 protein levels with TH287
	BJ cells with hTERT, hTERT/SV40LgT or hTERT/SV40LgT/RasV12	Human foreskin fibroblasts	TH287, TH588	-Cell viability and clonogenic survival	-Reduced cell viability and clonogenic survival in hTERT/SV40T and hTERT/SV40T/RasV12 cells, compared to hTERT cells
	HCT116 WT or p53 ^−/−^	Human colorectal cancer	TH287, TH588	-Cell viability	-No significant difference in viability between isogenic p53 WT and p53 ^−/−^ cells
	Patient Derived Xenograft	Metastases from patient with BRAF*^V600E^*-mutated melanoma, whose tumor was resistant to carboplatin/dacarbazine/vemurafenib	TH588, once daily, 30 mg/kg, for 20 days	-In vivo: TH588 subcutaneously injected in female NOD-SCID IL2Rγnull (NOG) mice, tumor growth monitored	-Significant reduction in tumor growth
	MCF7	Human breast adenocarcinoma	TH588, once daily, 30 mg/kg, for 18 days	-In vivo mouse xenograft: tumor volume measured	-Significant reduction in tumor volume
	SW480	Human colorectal cancer cell line (established from primary tumor)	-In vitro: siRNA #3, dox-inducible shMTH1, TH588 -In vivo: Dox inducible shMTH1 cells, TH588, once daily, 30 mg/kg, for 35 days	-Cell survival -Subcutaneous xenograft tumors in female SCID mice	-Reduced cell survival -Reduced mouse xenograft tumors, improved survival
	SW620	Human colorectal cancer cell line (from metastatic SW480 tumor)	siRNA # 3	-Cell survival	-Better survival under MTH1 siRNA vs. SW480 cells
	HeLa	Human cervical cancer	siRNA # 3	-Cell survival	-Low surviving fraction in siMTH1 cells
	HEK293T	Human embryonic kidney, SV40 T-antigen	siRNA # 3	-Cell survival	-Low surviving fraction in siMTH1 cells
	MB231	Human breast adenocarcinoma	siRNA # 3	-Cell survival	-Intermediate surviving fraction in siMTH1 cells
	LNCaP, DU145, PC-3	Human prostate cancer	siRNA # 3	-Cell survival	-Better survival in siMTH1 LNCaP and PC-3 cells vs. DU145
Huber et al., Nature, 2014 [36]	SW480	Human colorectal cancer	SCH51344, (S)-Crizotinib, shRNA, siRNA	-Viability, colony formation -Immunofluorescent staining for DNA damage markers: 53BP1 and phospho-S1981 ATM -MUTYH/OGG1 alkaline comet assay -SCID xenograft tumor model	-SCH51344 and (S)-Crizotinib decreased cell viability, increased 53BP1 and ATM autophosphorylation, increased comet tail moments. MTH1 overexpression reduced DSBs -MTH1 siRNA and shRNA impaired colony formation -No difference in viability between ATM or ATR inhibitor-treated shMTH1 and shGFP -(S)-Crizotinib significantly reduced xenograft tumors
	SW480 shp53 Tet-on system	Human colorectal cancer	SCH51344, (S)-Crizotinib	-Viability	-No significant difference in viability under p53 knockdown
	HCT116 WT, p53 ^−/−^, p21 ^−/−^	Human colorectal cancer	SCH51344, (S)-Crizotinib	-Viability	-SCH51344: no difference in viability between WT, p53 ^−/−^, and p21 ^−/−^ cells -(S)-Crizotinib: p21 ^−/−^ cells were strongly sensitized compared to WT and p53 ^−/−^ cells
	BJ cells: WT, hTERT, hTERT/SV40Lg T, or hTERT/SV40LgT/RasV12	Human foreskin fibroblasts	SCH51344, (S)-Crizotinib	-Viability	-Reduced cell viability in hTERT/SV40T and hTERT/SV40T/RasV12 cells, compared to hTERT or WT cells -Reduced viability with (S)-Crizotinib vs. SCH51344
	DLD1	Human colorectal cancer	siRNA	-Colony formation	-siMTH1 impaired colony formation
	ATM proficient vs. ATM ^−/−^ MEFs	Mouse embryonic Fibroblasts (MEFs)	SCH51344, (S)-Crizotinib	-Viability	-No difference in viability between ATM ^−/−^ and ATM-proficient MEFs
	PANC-1	Human pancreatic cancer	(S)-Crizotinib	-Colony formation	-Inhibition of colony formation
Warpman et al., Annals of Oncology 2016 [42]	U2OS	Human osteosarcoma	siRNA, TH1579	-Cell viability -Modified comet assay (with MutT, or OGG1, or *N*-acetyl cysteine) -Immunofluorescence for DNA damage foci: DNA-PK, XRCC1, γH2AX, 53BP1	-TH1579 effectively introduced 8-oxoGua into cells, and this effect was reversed with *N*-acetyl cysteine, overexpression of human MTH1 or bacterial MutT. Effect can be enhanced by treatment with OGG1. Increased DNA damage foci i.e., DNA-PK, XRCC1, γH2AX, 53BP1 was observed.
	SW480	Human colorectal cancer	TH1579, in vitro and in vivo. For in vivo model: once daily at 30 mg/kg, 60 mg/kg, or 90 mg/kg	-In vitro: Modified comet assay -In vivo: Xenograft mouse model, tumor volume and animal weight measured	-MTH1 overexpression mitigated comet taillength as measured by a modified comet assay, following TH1579 treatment. Comet tail moment increased with OGG1 treatment -Effective reduction in tumor growth
	SW480.SN3: derivative of SW480	Human colorectal cancer	shRNA	-In vitro: Cell viability -In vivo: injected doxycycline-inducible shMTH1 in female SCID mice, tumor volumes were measured	-Tumor formation in mice reflected/tracked with level of MTH1 knockdown in vitro
	BJ hTERT/SV40T/RasV12	Transformed human foreskin fibroblasts	TH1579	-Cell viability	-More cytotoxicity in BJ-hTERT-Ras-SV40T cells vs. non-transformed counterpart (BJ-hTERT)
	NTUB1/P	Human drug resistant bladder cancer	-TH588, TH816, IACS-4759, TH1579 -AstraZeneca (AZ) compounds **15**, **19**, **24** [38] -AZ siRNA [38]	-Chemical inhibitors of MTH1: 8-oxo-dG incorporation via immunofluorescence -AZ siRNA: Cell viability	-8-oxo-dG incorporation into the DNA correlated with MTH1-inhibition associated cytotoxicity -AZ siRNA: Resulted in loss of viability in NTUBP1/P cancer cells
	Patient Derived Xenograft	Metastatic carboplatin/dacarbazine/vemurafenib –resistant BRAF*^V600E^*-mutated melanoma,	TH1579, in vivo: 45 mg/kg daily, via oral gavage for approximately 40 days	-Tumor growth measured in mice	-Significant reduction in tumor growth
	HCT116	Human colorectal cancer	TH588, TH1579	-TH588 and anti-tubulin drugs: Proteomics profile -TH1579 in vitro: Immunoblot to assess phospho-p53 (S15), total p53, p21, cleaved PARP, cleaved caspase 3, γH2AX -TH1579 in vivo: Mouse xenograft experiment, 90 mg/kg. Measured 8-oxo-dG, number of 53BP1, γH2AX, caspase 3, Ki67 foci. Immunoblotting of mouse tumors for phospho-p53 (S15), total p53, p21, cleaved PARP, γH2AX levels	-Proteomics profile: TH588 clustered with nutrient starvation—Immunoblot in vitro TH1579 treatment: increased phospho-p53, total p53, p21, cleaved PARP, cleaved caspase 3, and γH2AX -In vivo TH1579 treatment: increased 8-oxo-dG, 53BP1 and caspase 3 foci. A decrease in Ki67 foci was observed. Immunoblot: elevated p53, small increase in phospho-p53, increase in p21, minor elevation in cleaved PARP levels, no noticeable change in γH2AX levels
Kawamura et al., Scientific Reports 2016 [37]	HeLa	Human cervical cancer	NPD7155, NPD9948, TH287, (S)-Crizotinib, SCH51344, MTH1 siRNA	-Cell viability -8-oxo-dG and 53BP1 foci measured via immunofluorescence -Proteomics profiling and tubulin polymerization -Cell cycle analysis -Immunoblot for Bcl-2	-NPD7155 and NPD9948 exhibited weak cytotoxicity, compared to TH287 and (S)-Crizotinib. NPD7155 and NPD9948 did not introduce 8-oxo-dG into DNA to the same extent as TH287. NPD7155 and NPD9948 induced 53BP1 foci at 100 µM -TH287 and TH588 inhibited tubulin polymerization, at 30 µM or higher -TH287 and TH588, but not NPD7155 and NPD9948, increased cells in G2/M phase, -TH287 and TH588 induced mild Bcl-2 phosphorylation, similar to tubulin-targeting agent Vinblastine (immunoblot for supershift in Bcl2 signal) -MTH1 siRNA did not affect cell survival or cell cycle progression
	PANC-1	Human pancreatic cancer	NPD7155, NPD9948, TH287, (S)-Crizotinib, SCH51344	-Cell viability	-100 µM, NPD7155: ~ 50% loss of viability -100 µM NPD9948: ~ 30% loss of viability -TH287: 10 µM, 70% reduction in viability -(S)-Crizotinib: 10 µM, ~20% reduction in viability -SCH51344: 10 µM, ~15% reduction in viability
	MIA PaCa-2	Human pancreatic cancer	NPD7155, NPD9948, TH287, (S)- Crizotinib, SCH51344	-Cell viability	-30 µM, NPD7155: ~25% loss of viability -30 µM, NPD9948: no loss of viability -TH287: 3 µM, 90% reduction in viability -(S)-Crizotinib: 10 µM, 70% reduction in viability -SCH51344: 10 µM, 30% reduction in viability
	NIH3T3 or NIH3T3/KRAS	Non-transformed MEFs, vs. KRAS transformed MEFs	NPD7155, NPD9948, TH287, (S)-Crizotinib, SCH51344	-Cell viability	-NPD7155: Small decrease in viability in NIH3T3/KRAS compared to NIH3T3 cells -Remaining MTH1 inhibitors: no significant difference in viability between NIH3T3/KRAS and NIH3T3 cells
	MG-63	Human osteosarcoma	NPD7155, NPD9948, TH287, (S)-Crizotinib, SCH51344	-Cell viability	-30 µM, NPD7155: ~20% loss of viability -30 µM NPD9948: 10% loss of viability -TH287: 3 µM, 90% reduction in viability -(S)-Crizotinib: 10 µM, 60% reduction in viability -SCH51344: 10 µM, ~25% reduction in viability
Kettle et al., Journal of Medicinal Chemistry, 2016 [38]	U2OS, A549, H358, MCF7	Human osteosarcoma, lung adenocarcinomas, breast adenocarcinoma	TH588, S-crizotinib, AZ compounds **15**, **19**	-Growth inhibition (GI50)	-TH588 and (S)-Crizotinib had low GI50s, in the range of 2 to 5 µM. Compounds **15** and **19** had high GI50s: >30 µM for compound **15**, and between 6 and 14 µM for compound **19**
	U2OS	Human osteosarcoma	TH588, compound **19**, commercially available siRNA, siRNA # 3 from Gad et al., 2014 [35]	-TH588, compound 19: DNA damage response (DDR) signaling markers via immunoblot: p-Ser1981 ATM, p-Ser15 p53, γH2AX, RPA, cleaved PARP1 -Cell viability	-Elevated p-Ser15p53, total p53, cleaved-PARP1 with TH588. No effect on DDR signaling activation or apoptosis with compound **19**. Neither compound changed γH2AX or RPA levels. -Commercially available siRNA did not affect cell viability, siRNA # 3 strongly impaired cell viability -TH588, (S)-Crizotinib, and compound 19 killed MTH1 siRNA transfected cells
	SW480	Human colorectal cancer	CRISPR-mediated knockout	-Cell viability	-Clones with complete MTH1 knockout did not show impaired growth relative to parental MTH1 WT cells -(S)-Crizotinib and compound **19** killed MTH1 knockout and parental cells equivalently
Petrocchi et al., Bioorg. Med. Chem. Lett., 2016 [39]	U2OS, SaOS2, SW480, MDA-MB-231, HeLa, UOK262, 293T, A549, H460, H358, WI38, BJ, hMEC	Human cell lines (osteosarcoma, colorectal/breast/cervical cancer, embryonic kidney, lung cancer, fetal lung and foreskin fibroblasts, mammary epithelial	IACS-4759, IACS-4619	-Cell viability	-No cytotoxicity at concentrations up to 50 µM. Data not shown
